# Home-built environment interventions and inflammation biomarkers: a systematic review and meta-analysis protocol

**DOI:** 10.3399/BJGPO.2022.0104

**Published:** 2022-10-19

**Authors:** Eva Hernandez-Garcia, Evangelia Chrysikou, Larissa Nekhlyudov, Derek W Gilroy, José M Ordóñez-Mena

**Affiliations:** 1 The Bartlett School of Sustainable Construction, University College London, London, UK; 2 Clinic of Social and Family Medicine, Department of Social Medicine, University of Crete, Heraklion, Greece; 3 Department of Medicine, Brigham and Women’s Hospital, Harvard Medical School, Boston, MA, USA; 4 Department of Medical Oncology, Dana-Farber Cancer Institute, Harvard Medical School, Boston, MA, USA; 5 Centre for Clinical Pharmacology and Therapeutics, Division of Medicine, University College London, London, UK; 6 Nuffield Department of Primary Care Health Sciences, University of Oxford, Oxford, UK

**Keywords:** housing, home-built environment, biomarkers, inflammation, cancer survivor, comorbidity, chronic disease, general practice, community care

## Abstract

**Background:**

Inflammation control is a fundamental part of chronic care in patients with a history of cancer and comorbidity. As the risk–benefit profile of anti-inflammatory drugs is unclear in survivors of cancer, GPs and patients could benefit from alternative non-pharmacological treatment options for dysregulated inflammation. There is a potential for home-built environment (H-BE) interventions to modulate inflammation; however, discrepancies exist between studies.

**Aim:**

To evaluate the effectiveness of H-BE interventions on cancer-associated inflammation biomarkers.

**Design & setting:**

A systematic review and meta-analysis of randomised and non-randomised trials in community-dwelling adults.

**Method:**

PubMed and MEDLINE, Embase, Web of Science, and Google Scholar will be searched for clinical trials published in January 2000 onwards. The study will include H-BE interventions modifying air quality, thermal comfort, non-ionising radiation, noise, nature, and water. No restrictions to study population will be applied to allow deriving expectations for effects of the interventions in cancer survivors from available source populations. Outcome measures will be inflammatory biomarkers clinically and physiologically relevant to cancer. The first reviewer will independently screen articles together with GPs and extract data that will be verified by a second reviewer. The quality of studies will be assessed using the Cochrane risk-of-bias tools. Depending on the clinical and methodological homogeneity of populations, interventions, and outcomes, a meta-analysis will be conducted using random-effects models.

**Conclusion:**

Findings will determine the effectiveness of H-BE interventions on inflammatory parameters, guide future directions for its provision in community-dwelling survivors of cancer and support GPs with safer anti-inflammatory treatment options in high-risk patients for clinical complications.

## How this fits in

Provision of treatment options for inflammation control is a fundamental component for the management of common chronic diseases in primary and community care, especially of the complex medical and pathophysiological profile of survivors of cancer. The most compelling evidence for an association comes from randomised controlled trials that test drugs or exercise–nutritional programmes aimed at modulating inflammatory response. While non-steroidal anti-inflammatory drugs and systemic glucocorticoids are frequently prescribed in general practice, the effects are still controversial in survivors of cancer as these may be unsafe and hinder restoring the normal regulation of inflammation. The potential benefit of H-BE interventions on cancer-associated inflammation biomarkers may mean they are a reasonable treatment to improve quality of life and clinical outcomes in community-dwelling older patients and ultimately in survivors of cancer.

## Introduction

Cancer survival in high-income countries continues to improve across almost all cancer types, even for those with the worst prognosis.^1,2^ The delivery of multiple evidence-based interventions has been an important driver of the progress in cancer control, particularly around the management of comorbidities.^2^ Compared with the cancer-free population, survivors of cancer are at significantly higher risk for mental health,^3–5^ cardiometabolic, musculoskeletal,^6–8^ somatic, and physical conditions^9–11^ years after primary treatment. Significant predictors of the number of comorbidities post-diagnosis include cancer type, treatment received, years since diagnosis, age, adiposity, physical activity, and level of deprivation.^12–20^ Unique multimorbidity clusters drive differences on cancer survival outcomes,^17,18^ drug prescriptions,^19^ GP contacts and home visits,^21,22^ and hospitalisations.^11^

Inflammatory biomarkers are postulated to derive a clinically relevant metric in the early prediction of multimorbidity, including diseases of various physiologic systems.^23,24^ Combined inflammatory markers have shown to predict treatment response,^25–27^ early recurrence,^28,29^ prognosis,^30^ and comorbidity development after cancer diagnosis.^31^ In primary care settings, prediction models for cancer that include inflammatory biomarkers demonstrate superior clinical utility compared with symptoms-only scores.^32^ GPs commonly request blood test combinations that check for abnormal inflammation in patients such as C-reactive protein (CRP), full blood count, glycated haemoglobin, ferritin and/or neutrophil count.^33,34^ Their usefulness as surrogate endpoints has been confirmed in clinical trials in multiple cancer types.^35^

Improvements to the H-BE lead to better health.^36^ Housing refurbishment of new energy-efficient combination (combi) boilers and double-glazed windows in social housing showed a reduction of 16% in healthcare service utilisation costs over 6-months and improved the residents’ health status, particularly in people aged ≥65 years.^37^ Multiple home improvements — electric systems, windows, wall insulation, and garden paths — to meet UK housing quality standards were associated with up to 35% and 52% fewer emergency admissions for cardiovascular and respiratory conditions among all-aged residents within a 10-year period compared with people who did not receive the intervention.^38^ Interventions modifying the household environmental quality — air, artificial lighting and nature — trigger changes on the residents’ inflammatory levels, particularly interleukin-6, CRP, high sensitivity-CRP, endothelial growth factor, granulocyte-colony stimulating factor, and eotaxin.^39^

**Figure 1.**
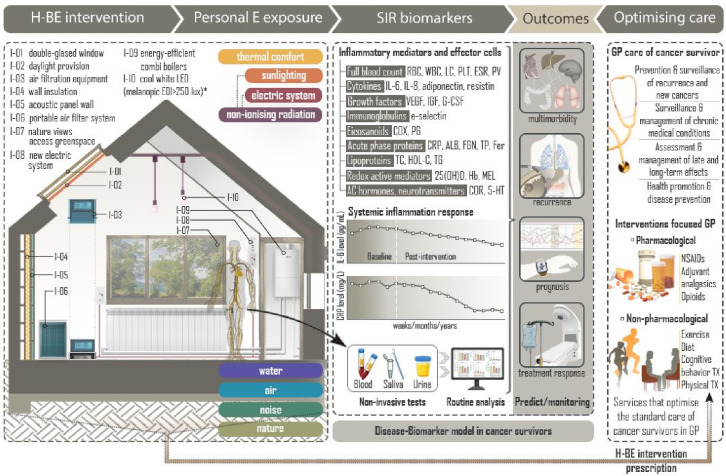
Conceptual framework describing the relationships between home-built environment intervention, systemic inflammatory response, clinical outcomes and general practice care of survivors of cancer. ^*^In daytime. Minimum melanopic equivalent daylight illuminance at the eye measured in the vertical plane at 1.2m height. 25(OH)D = 25 hydroxyvitamin D. 5-HT = 5-hydroxytryptamine/serotonin. AC = adrenal cortex. ALB = albumin. COR = cortisol. COX = cyclooxygenase. CRP = c-reactive protein. E = environmental. EDI = melanopic equivalent daylight illuminance. ESR = erythrocyte sedimentation rate. Fer = ferritin. FGN = fibrinogen. G-CSF = granulocyte-colony stimulating factor. GP = general practice. Hb = haemoglobin. H-BE = home-built environment. HDL-C = high-density lipoprotein cholesterol. IGF = insulin-like growth factor. IL = interleukin. LC = leukocyte count. LED = light-emitting diode. MEL = melatonin. NSAIDs = non-steroidal anti-inflammatory drugs. Personal E exposure = Personal environmental exposure. PG = prostaglandin. PLT = platelets. PV = plasma viscosity. RBC = red blood cells. SIR = systemic inflammatory response. TC = total cholesterol. TG = triglycerides. TP = total protein. TX = therapy. VEGF = vascular endothelial growth factor. WBC = white blood cells.

This systematic review will examine the effectiveness of H-BE interventions modifying air quality, thermal comfort, non-ionising radiation, noise, nature, and water on inflammatory biomarkers in community-dwelling adults. This study is intended to provide the groundwork for future H-BE interventions as inflammation-targeting treatment in survivors of cancer for potential consideration in general practice (Figure 1).

## Method

This review will be developed according to the Preferred Reporting Items for Systematic Reviews and Meta-Analyses (PRISMA) statement.^40^ This protocol conforms with the PRISMA-Protocols checklist^41^ and is registered with PROSPERO (CRD42022310680).

### Eligibility criteria

#### Types of study

All types of clinical trials will be included (randomised and pseudo-randomised controlled and uncontrolled trials, and so on) if these were published, peer reviewed, and reported primary research and quantitative data. Trials will refer to experimental or interventional studies in which the researchers intervened to modify the H-BE. Mixed-methods studies employing quantitative data will be included if meeting the inclusion criteria.

The operational definition of clinical trial and further discussion on the eligible study types is reported in Supplementary Box 1.

#### Participants and setting

Given the limited studies addressing the research question in survivors of cancer, no restrictions will be applied to participants other than those applied by the included primary publications itself. This is intended to avoid missing relevant data that identify the interactions between H-BE interventions and inflammation, and translate the potential benefits to survivors of cancer. Thus the study will include adults (aged ≥18 years) living in the community at any H-BE as their place of usual residence. The term H-BE is described in the Supplementary Box 2.

#### Interventions

A H-BE intervention is defined as any change of baseline housing conditions for a modified environmental quality by using architectural elements or devices. Architecture elements are referred to as a new installation, supply, or retrofitting related to any building physical characteristic, design configurations, and engineering system within homes ; for example, double-glazing of windows and air filtration. Household environmental quality will be considered — air quality, thermal comfort, non-ionising radiation, noise, nature, and water — and will be monitored using quantitative measurement equipment (Supplementary Table 1). Potential H-BE interventions by environmental exposure category are described in the Supplementary Table 2. No restrictions will apply to interventions in terms of delivery, dose, duration, intensity, frequency, and co-interventions.

#### Comparison

Studies with or without any comparative group will be considered for the review.

#### Outcome measures

The outcome will be inflammatory biomarkers in blood, urine, and saliva, either examined individually or in combination as part of a score. A comprehensive review of clinical and preclinical data was done to identify the cancer-associated inflammation markers for this study (Table 1; Supplementary Table 3).

**Table 1.** Summary of cancer-associated systemic inflammatory response biomarkers

Group	ID	Marker	ID	Marker
Circulating individual inflammatory markers^a^				
G1–10	Inflammatory mediators			
G1	Cytokines			
	1–42	Interleukins	94–96	Interferons
	43–47	Colony-stimulating factors	97–99	Tumour necrosis factor
	48–51	Adipokines	100	Macrophage migration inhibitory factor
	52–93	Chemokines		
G2	Growth factors			
	101	Transforming growth factor	112	Hepatocyte growth factor
	102–104	Vascular endothelial growth factor	113	Nerve growth factor
	105	Platelet-derived growth factor	114,115	Insulin, insulin-like growth factor
	106–108	Fibroblast growth factor	116–118	Endothelins
	109,110	Epidermal growth factor	119,120	Renin-angiotensin system
	111	Placental growth factor	121–129	Angiopoietin, angiopoietin-like protein
G3	Transcription factors			
	130	Nuclear factor kappa B	133–138	Signal transducers and activators of transcription
	131,132	Nuclear factor erythroid 2-related factor	139–141	Hypoxia-inducible factor
G4	Immunoglobulins			
	142–149	Cell-adhesion molecules	150–152	Programmed cell death protein
G5	Eicosanoids			
	153,154	Cyclooxygenase	160	Lipoxygenase
	155–158	Prostaglandins	161–164	Leukotrienes
	159	Thromboxane	165	Lipoxins
G6	Acute phase proteins			
	166–168	C-reactive protein	188–195	Plasminogen activation system
	169–172	Pentraxins family	196–197	Microglobulins
	173–175	Serum amyloid A	198–201	Transport proteins
	176–178	Alpha globulins	202–205	Complement system
	179–184	Extracellular matrix proteins	206–209	Albumin, transferrin
	185–187	Fibrinogen, D-dimer		
G7	Matrix metalloproteinases			
	210–225	Matrix metalloproteinases		
G8	Redox active mediators			
	226,227	Metalloproteins (haemoglobin; heme)	233	Calcitriol
	228–231	Vitamin D (25-hydroxyvitamin D)	234	Melatonin
	232	Calcidiol	235	6-sulfatoxymelatonin
G9	Lipoproteins			
	236–238	Very low-, low-, high-density lipoprotein	241	Total cholesterol
	239	Oxidised low-density lipoprotein	242	Triglycerides
	240	Apolipoprotein		
G10	Adrenal cortex hormones and neurotransmitters			
	243–245	Glucocorticoids	251–253	Catecholamines
	246–250	Neurotransmitters		
G11–13	Inflammatory effector cells			
	254	Platelets	259–265	White blood cells
	255–258	Red blood cells		
Combining multiple inflammatory markers (into a score)^a^				
cG11.13	White blood cells-platelets parameters			
	266	Lymphocyte to monocyte ratio	269	Derived Neutrophil to lymphocyte ratio
	267	Neutrophil to lymphocyte ratio	270–272	Novel combined scoring system^b^
	268	Platelet to lymphocyte ratio		
cG6	Acute phase proteins parameters, combinations			
	273–275	Glasgow prognostic scores	277–280	Novel combined scoring system^b^
	276	Prognostic inflammatory and nutritional index		
cG11.6	White blood cells-acute phase proteins parameters, combinations			
	281–285	Novel combined scoring system^b^		
cG11.6.8	White blood cells-acute phase proteins-redox active mediators parameters			
	286	Combined haemoglobin, albumin, lymphocyte, platelet		
cG10	Lipoprotein particle-derived measure of insulin resistance			
	287	Lipoprotein insulin resistance score		

^a^The proposed panel of 287 cancer-associated inflammatory biomarkers could be modified and upgraded over time in accordance with clinical efficacy tested and promising clinical results of novel candidates. Before eligibility, it was verified that each biomarker could be identified in blood, urine, or saliva samples.^b^Novel combined inflammation-based scoring systems proposed in further research will be incorporated into panel.

G = group. cG = combined group. ID = identifier

### Information sources

Articles will be sought using PubMed and MEDLINE, Embase, Web of Science databases, and Google Scholar.^42–44^ Additional non-indexed citations will be identified by handsearching and scrutiny of reference lists of eligible studies to minimise potential reporting bias.^45^ The authors will also retrieve registered clinical trials from Cochrane Central Register of Controlled Trials, International Clinical Trials Registry Platform, and ClinicalTrials.gov that are not indexed in bibliographic databases.^46,47^

### Search strategy

The search algorithm combines the terms 'home settings' and 'environmental attributes' and 'inflammatory biomarkers' using Boolean operators, truncation, and proximity operators. Controlled vocabulary terms and free-text words were identified and refined through an iterative process of preliminary searches in databases and snowballing technique. The initial search strategy developed for PubMed (Supplementary Table 4) will be tailored appropriately as required for each database. Harzing’s Publish or Perish software (version 7)^48^ will be used to retrieve the first collated 300 records titles from Google Scholar.^49^ Databases will be searched for the period 1 January 2000 onwards, since investigations that address H-BE interventions and inflammatory biomarkers are scarce before this timeframe.

There will be no filtering for study design as these may not achieve sufficient sensitivity and miss potential studies.^50,51^ Validated search query filters for Humans will be added to the final search algorithm as the *Cochrane Handbook for Systematic Reviews of Interventions* recommends.^52^ Given the non-English language studies rarely impact on the effect estimates and conclusions of systematic reviews and meta-analyses,^43,53^ these will be only labelled as 'studies awaiting classification' in the PRISMA flow diagram to inform its availability.

### Data records, management, and extraction

All records identified will be stored in Mendeley software. The Systematic Review Assistant-Deduplication Module will be used to automatically remove duplicated references,^54^ and the screening process will be undertaken in Rayyan tool.^55,56^

One review author (EHG) will independently screen titles and abstracts of records in duplicate with a GP group, crowdsourcing citation-screening.^57^ Identified articles will be randomly split among the GPs involved (≤300 articles each). If studies remain, these will be distributed among the review team or a second reviewer(s). The same method will be used to screen the full-text (≤3 articles each GP). If no abstract or not enough information is available, the study will be retained for full-text screening. Discrepancies will be resolved through discussion until consensus is reached.

Data will be extracted by one reviewer (EHG) and verified by a second reviewer for quality assurance. A predefined data-extraction form will be initially developed using Cochrane^58^ and JBI manuals,^59^ including study information, methodology, participant characteristics, interventions, and outcomes (Supplementary Table 5). Whenever necessary, the corresponding author will be contacted by email to request information.

#### Involvement of GPs

The process of study selection will be done in collaboration with non-academic GPs, adopting the National Institute for Health Research involvement activity framework.^60^ Despite the validity of crowdsourced citation-screening by untrained workers,^57^ additional quality assurance tests will be conducted as part of this review to improve confidence in the results.^61^ The strategy for the involvement and inclusion criteria of GPs and quality control mechanisms in crowdsourcing are outlined in Supplementary Box 3.

The Guidance for Reporting Involvement of Patients and the Public version 2 (GRIPP2) will be used to ensure the overall quality and transparency of the involvement activity in this research.^62^

### Methodological quality assessment

#### Risk-of-bias assessment

The risk of bias will be assessed with the Cochrane risk-of-bias tool for randomised controlled trials (RoB 2 RCTs), across several features of trial design, management, and reporting.^63^ Additionally, the test version of the RoB 2 tool will be used for crossover trials with specific considerations required in this study design.^64^ Judgement is assigned as 'Low' or 'High' risk-of-bias, or 'Some concerns'.

For the other experimental studies, controlled or uncontrolled trials, the Cochrane risk of bias in non-randomised studies of interventions (ROBINS-I) guideline will be used.^65,66^ Bias domains include confounding, participant selection, classification of the interventions, deviations from intended interventions, missing data, outcome measurements, and reported results. Judgement is classified as 'Critical', 'Serious', 'Moderate', and 'Low' risk of bias.

ROBINS-I tool is frequently misapplied in practice.^67^ The risk-of-bias assessment will be performed by the first reviewer (EHG) and a random sample will be verified by the review member with methodological expertise (JMOM) to ensure that they do not disregard more intricate domains of bias.

#### Quality assessment

The quality of evidence for an association between intervention(s) and inflammatory biomarker concentrations will be rated using the Grading of Recommendations Assessment, Development, and Evaluation (GRADE) approach across five domains: study limitations, imprecision, indirectness, inconsistency, and publication bias.^68^ The relevant risk-of-bias tool will be integrated within GRADE assessment and it will be accepted that both randomised and non-randomised experimental studies are the reference initial for highest feasible certainty.^69^

### Data synthesis

The findings will be reported narratively and supplemented with summary tables structured by the type of intervention. The criteria used to prioritise the reporting results will be based on the type of study design and will be separate for randomised, pseudo-randomised, and crossover trials (RoB 2) and other experimental studies controlled or uncontrolled trials (ROBINS-I, which has a separate domain to address confounding). To enhance transparency in reporting the quantitative effects of H-BE interventions, the Synthesis Without Meta-analysis (SWiM) guideline will be followed.^70^

### Statistical analysis

If inflammatory biomarkers are reported at the end of the study or as a change from baseline, raw or adjusted unstandardised mean differences with a 95% confidence interval (CI) will be extracted or calculated to compare intervention and comparator arms. If means and standard deviations are not available, these will be calculated from medians and interquartile ranges using Wan *et al*’s equations.^71^ If inflammatory biomarkers are reported as below or above a certain threshold, as categorical outcomes, raw or adjusted odds ratio or risk ratios will be extracted or calculated with 95% CI. Random-effects models will be used to pool study-specific effect size measures using the Paule and Mandel estimator for the between-study variance.^72^

The authors' previous analysis on the topic^39^ showed that studies reported multiple or single biomarker(s) at multiple time points. Therefore, subgroup analyses will be conducted by length of follow-up: short term (≤2 weeks), mid-term (>2 weeks to ≤6 months), and long term (>6 months).

The robustness of the findings will be evaluated with sensitivity analyses (excluding studies at high risk of bias).

The *I* ^2^ statistic will be computed with 95% CIs to quantify the proportion of heterogeneity not attributable to sampling error. The Cochrane thresholds of *I* ^2^ will be used for unimportant heterogeneity (0%–40%); moderate (30–60%); substantial (50–90%) and, considerable heterogeneity (>75%).^73^ If the number of studies is small in the meta-analysis, the *I* ^2^ statistic will be interpreted cautiously as it can be biased owing to low statistical power.^74^ The significance of the heterogeneity will be tested with the χ^2^ test.^73^ Statistical significance will be set at *P*<0.05.

Publication bias will be evaluated using Begg and Mazumdar’s funnel plot^75^ and the Egger’s linear regression test.^76,77^

Analysis will be conducted using statistical software of R,^78^ with the R packages meta and metafor.^79^

## Discussion

### Summary

This systematic review of clinical trials will provide insights on the effectiveness of H-BE interventions on reducing inflammatory parameters of community-dwelling adults, the quality of the evidence provided by these studies, and their reliability to inform the potential adoption by GP surgeries, clinical commissioning groups, and patients themselves.

### Strengths and limitations

Given survivors of cancer are vastly underrepresented in this research area,^39,80^ the generalisability of the findings from ‘adults’ to ‘survivors of cancer’ will be considered.^81^ Biomarker endpoints that are physiologically relevant to disease pathology and reflect earlier phase of disease progression are a useful approach to support extrapolation.^81,82^ A panel of inflammatory biomarkers was derived from clinical and preclinical research. These biomarkers have been shown to predict comorbidity development, treatment response, recurrence, and prognosis in survivors of cancer. Cancer-associated inflammatory mediators from preclinical data are also relevant since there is biologically plausibility to treat them as surrogate endpoints in clinical trials.

While this systematic review may not generate immediate recommendations for clinical practice that are specific to survivors of cancer, evidence from high quality RCTs will generate meaningful information about the effects of H-BE interventions on the systemic inflammatory responses. The findings may identify promising H-BE interventions that will need further investigation in trials with long-term non-surrogate hard outcomes and multimodal treatment programmes. As a major limitation, clinical and methodological heterogeneity between studies is anticipated, with different study designs, populations included, interventions administered, and outcome definitions; as such, appropriate interpretation of results will require caution. Another weakness is that the assessment of risk of bias will only be conducted in duplicate in a random sample of included studies.

### Implications for research and practice

Cancer treatments lead to long-lasting immune dysfunction, chronic non-resolving inflammation,^83,84^ increased comorbidity burden,^31^ and epigenetic age acceleration associated with an elevated inflammatory profile.^85^ Interventions to mitigate inflammation may benefit survivors of cancer. In general practice, non-steroidal anti-inflammatory drugs are widely prescribed.^86,87^ However, their regular use on cancer course is still controversial, ranging from promising chemopreventive effects of aspirin use;^88,89^ to little or no effect of celecobix use on cancer recurrence, progression, and death; and cardiovascular toxic effects.^90,91^ While the effect of glucocorticoids as anti-inflammatory agents on survival outcomes remains inconclusive,^92^ the steroid regimen administered may cause long-term adverse metabolic events.^93^ Against this, GPs are calling for alternative treatments to the routine use of anti-inflammatory drugs in patients with comorbidities, including non-pharmacological therapies.^94^ Treatment options promoting pro-resolution processes of inflammation may be superior to standard anti-inflammatory strategies.^95^

One potential non-pharmacological area for intervention is the H-BE. There is, therefore, a need for evidence-based information around which H-BE interventions are effective to improve inflammation-related outcomes and what available knowledge translation tools could efficiently support its delivery. This study will provide further understanding of H-BE interventions as potential therapeutics for inflammation control. Given that the knowledge is scarce around care through H-BE for survivors of cancer,^80^ findings will serve as a resource for a potential applied research field in survivors of cancer and for which interventions may be implemented into primary care. Thus, this review may support GPs against the increasing demand of other safer inflammation-modulating treatment options, especially when considering prescribing anti-inflammatory drugs in patients at risk of clinical complications. Overall, primary care professionals and researchers may optimise the standard chronic care by understanding this evidence for and against their use.

## Data

The dataset on which the conclusions of the proposed research will rely can be obtained from the corresponding author on reasonable request.

## Trial registration number

PROSPERO: CRD42022310680.
